# Pocket closure after repeated subgingival instrumentation: a stress test to the EFP guideline for stage III-IV periodontitis

**DOI:** 10.1007/s00784-023-05279-6

**Published:** 2023-09-29

**Authors:** Francesco Ferrarotti, Giacomo Baima, Martina Rendinelli, Filippo Citterio, Giulia Maria Mariani, Federico Mussano, Federica Romano, Mario Romandini, Mario Aimetti

**Affiliations:** 1 Department of Surgical Sciences, C.I.R. Dental School, Section of Periodontology, University of Turin, Via Nizza 230, Turin, Italy; 2 Politecnico di Torino, Turin, Italy; 3 Department of Periodontology, Faculty of Dentistry, University of Oslo, Geitmyrsveien, 69, 0455 Oslo, Norway

**Keywords:** Clinical practice guideline, Multilevel analysis, Nonsurgical periodontal debridement, Periodontal diseases, Periodontal pocket, Repeated subgingival instrumentation

## Abstract

**Objectives:**

To study the effects of one or two repeated subgingival instrumentations (RSI) in achieving the endpoints of therapy (EoT) in open pockets [residual probing pocket depth (PPD) ≥ 6 mm and PPD 4–5 mm with bleeding on probing (BoP)] after steps I-II of therapy.

**Materials and methods:**

Twenty-five patients (3,552 total sites; 1,450 open pockets) with stage III-IV periodontitis received steps I-II of periodontal therapy and were re-evaluated after 4–6 weeks (T1). Residual pockets received RSI at T1 and at 3 months (T2). EoT (PPD < 4 or PPD < 6 BoP-) rate at T1, T2 and 6 months (T3) was computed. The number of needed surgeries and treatment costs were calculated.

**Results:**

At T1, 67.6% of open pockets achieved EoT. At residual PPD ≥ 6 mm at T1 (*n* = 172), one and two RSI resulted in 33.1% and 45.9% of EoT at T2 and T3, respectively. At residual PPD 4–5 mm with BoP at T1 (*n* = 298), one and two RSI resulted in 66.8% and 72.1% of EoT at T2 and T3, respectively. PPD at T1 predicted EoT after RSI in both cases, while tooth type only in residual PPD 4–5 mm BoP + . At T1, mean number of surgeries per patient and associated costs were significantly higher than after one/two RSI.

**Conclusions:**

RSI may achieve EoT in residual PPD 4–5 mm BoP + and PPD ≥ 6 mm in a considerable number of cases.

**Clinical relevance:**

These findings may support the administration of one/two cycles of RSI prior to surgical approach.

**Protocol registration:**

ClinicalTrials.gov identification number: NCT04826926.

**Supplementary Information:**

The online version contains supplementary material available at 10.1007/s00784-023-05279-6.

## Introduction

Periodontitis represents one of the most common non-communicable diseases of humankind [1], with detrimental effects on both tooth-supporting structures and general health [2–5]. In late 2019, the European Federation of Periodontology (EFP) S3 level clinical practice guideline for the treatment of stage I-III periodontitis was approved by an international consensus of experts [6]. This guideline recommends a stepwise approach involving a first step aimed at controlling local and systemic risk factors, followed by a second step aimed at removing the subgingival biofilm through non-surgical instrumentation with or without adjunctive therapies [7]. After these steps, patients undergo a clinical re-evaluation to assess the achievement of the endpoints of therapy [EoT: probing pocket depth (PPD) < 4 or PPD < 6 with bleeding on probing (BoP)]. Residual PPD ≥ 6 mm represents indication for periodontal surgery; while PPD 4–5 mm with BoP indicates the need for subgingival re-instrumentation (RSI) [6, 8]. Conversely, sites achieving EoT are enrolled in the supportive periodontal care (SPC) recall system [9].

To this regard, it is still debated whether all residual PPD ≥ 6 mm should undergo surgery, since it has been shown that specific patients, teeth or sites may achieve the EoT after RSI [10, 11]. Moreover, it remains unclear to what extent residual PPD 4–5 with BoP achieves EoT after a second/third cycle of re-instrumentation. This is relevant because residual pockets during SPC have higher risk of disease progression and tooth loss [12, 13]. Finally, it is yet to be established whether open pockets (PPD ≥ 6 mm and PPD 4–5 mm with BoP) which achieved EoT at the first re-evaluation may remain closed at early SPC appointments [14].

Since the number of periodontal surgeries is strictly dependent on the distribution of residual pockets at the time of re-evaluation [8], it is relevant to understand to what extent is appropriate to stress on RSI for both economical and biological costs justification. Moreover, it would be crucial to understand which patients, teeth and sites possess the highest likelihood of EoT after RSI, and which are better indicated for an early surgical approach [15].

Therefore, the aims of this study were to assess: 1) the efficacy of RSI in achieving EoT in residual PPD ≥ 6 mm and 2) in sites 4–5 mm with BoP; 3) whether sites which achieved EoT at the first re-evaluation remained closed during early SPC appointments; 4) the factors at patient-, tooth- and site- level that can predict EoT at each different time point; 5) the need for surgery and the associated costs after one and two cycles of RSI.

## Materials and methods

This prospective clinical study was approved by the human subjects ethics board of “A.O.U. Città della Salute e della Scienza” of Turin (Protocol number: 0112880) and was conducted in accordance with the Helsinki Declaration of 1975, as revised in 2013. The manuscript is reported according to the STrengthening the Reporting of OBservational studies in Epidemiology (STROBE) guidelines [16].

### Study population

Stage III-IV periodontitis patients [4] were consecutively enrolled at the Section of Periodontology at the C.I.R. Dental School of Turin during the period between April 24^th^ and July 31^st^ 2021. All participants gave written informed consent. Age < 18 years, pregnancy or lactation, heavy smokers (> 10/day), conditions or diseases influencing periodontal healing (including uncontrolled diabetes mellitus), and the inability to complete any of the follow-ups were all considered exclusion criteria.

### Interventions

As part of the step I of treatment, all patients received oral hygiene instructions and motivation (OHI), and supragingival instrumentation was carried out in one single session. As for step II, subgingival instrumentation was performed using the quadrant-wise conventional method over a 2-week period by one experienced dental hygienist (MRe), using both ultrasonic devices (EMS Piezon Master, EMS, Nyon, Switzerland) and hand curettes (Gracey curettes, Hu-Friedy, Chicago, IL, USA) [7]. Chlorhexidine 0.12% rinses were prescribed twice a day for 1 min throughout the whole step II period [17]. As part of the study protocol, patients were recalled at 4–6 weeks (T1), 3 (T2) and 6 (T3) months after the end of step II. At any of these follow-up recalls, OHI were reinforced, and PPD = 4–5 mm with BoP as well as PPD ≥ 6 mm were re-instrumented using hand/ultrasonic instruments.

### Periodontal examination

A full-mouth periodontal examination was performed at baseline (T0), T1, T2 and T3 by the use of a manual periodontal probe (PCP-UNC15, Hu-Friedy, Chicago, IL, USA) by a single experienced examiner (FF). The following clinical parameters were recorded at 6 sites per tooth excluding third molars: PPD, recession (REC), clinical attachment level (CAL), BoP [18], and presence of plaque [19]. Moreover, mobility (Miller index) and furcation involvement were recorded. Full-mouth plaque (FMPS) and bleeding scores (FMBS) were calculated. All patients also received a full-mouth intraoral radiographic examination at T0 [4].

### Outcome measures

The primary outcome of this study was EoT defined at site level as PPD < 4 or PPD < 6 without BoP, evaluated at T1, T2 and T3. EoT was only evaluated on a subset of the available sites (*n* = 1,749) considered open at T0 (i.e., PPD ≥ 4 with BoP or PPD ≥ 6 mm). Sites experiencing relapse after EoT (T0 sites which achieved EoT at T1 and re-opened again) were also considered.

Secondary outcome variables included PPD, CAL and REC changes at the same time-points, evaluated considering all the sites (*n* = 3,726) of the included subjects. Furthermore, at each timepoint the number of surgeries needed as recommended by the treatment guideline was evaluated [6]. The number of surgeries was computed based on number and distribution of residual PPD ≥ 6 mm at T1, T2 and T3. When such residual pockets were present at the same sextant, they were considered as one single surgery. Also, the cost of the necessary procedures was studied using a fee schedule derived as an average from the tariff quota suggested by the National Association of Italian Dentists (ANDI).

### Sample size calculation

Sample size calculation has been performed using an online software. A previous report showed that the mean number of closed pockets at 2 months after non-surgical therapy was 52.5 ± 2.5 [20]. Thus, a further increase of 25% has been considered clinically relevant in order to justify the longer time of follow-up before the surgical treatment plan execution. To obtain a power of 80% and alpha 0.05, a sample of 23 patients have been considered adequate. In order to account for a possible 10% of drop-outs 25 patients were required.

### Statistical analyses

All the analyses were performed using a statistical software (STATA/SE®; Statacorp, College Station, Texas, US). Firstly, the rate of EoT at T1, T2 and T3 was described. Secondly, possible T1 predictors (i.e., PPD, plaque, tooth type, site location, mobility, furcation involvement) of EoT at T2 and T3 were explored through multilevel analysis adjusted for clustering. A priori subgroup analyses by residual PPD at T1 (PPD ≥ 6 mm; PPD 4–5 mm with BoP) were also reported. *P* < 0.05 was considered as statistically significant.

## Results

Data retrieved from 25 patients who completed the 6-month re-evaluation were included in the final analysis. Baseline characteristics of the study sample are presented in Table 1. On average, participants were middle aged (57.4 ± 7.9 years of age), 60.0% were males, with current light smokers being 40.0%. Participants presented a baseline mean CAL of 5.2 ± 2.5 mm, PPD of 4.0 ± 1.7 mm, and 71.1 ± 26.9 open pockets. FMBS and FMPS were 63.5 ± 23.0% and 73.9 ± 15.8%, respectively.

**Table 1:** Demographic and periodontal characteristics of included participants at baseline

Patient related variables	*n* = 25
Gender, N male (%)	15 (60.0)
Age (mean ± SD)	57.4 ± 7.9
Smokers, N (%)	10 (40.0)
Stage III/IV, N (%)	15/10
Grade A/B/C, N (%)	0/13/12
FMPS % (mean ± SD)	73.9 ± 15.8
FMBS % (mean ± SD)	63.5 ± 23.0
Open pockets (mean ± SD)	71.1 ± 26.9
Tooth related variables	*n* = 592
Tooth type (single/multi-rooted)	372/220
Mobility (0/1/2/3)	506/76/5/5
Site related variables	*n* = 3,552
PPD (mean ± SD)	4.0 ± 1.7
CAL (mean ± SD)	5.2 ± 2.5
REC (mean ± SD)	1.2 ± 1.5
Open pockets, N (%)	1,450 (40.8)
BoP positive, N (%)	2,199 (61.9)
Presence of plaque, N (%)	2,657 (74.8)
Furcation involvement (0/1/2/3)	1,211/31/64/14

*Abbreviations*: *BoP* bleeding on probing, *FMPS* full-mouth plaque score, *FMBS* full-mouth bleeding score, *PPD* probing pocket depth, *CAL* clinical attachment level, *REC* recession, *SD* standard deviation

### Clinical outcomes after single and repeated subgingival instrumentation

All patients were compliant with the study protocol. As expected, steps I-II of periodontal therapy resulted in a significant improvement of all clinical parameters at patient-level (Table 2). At T1, 18 teeth were extracted and a mean reduction of 28.5 ± 14.5 in the number of open pockets was achieved, together with a significant reduction in FMBS and FMPS. RSI provided an additional, albeit modest reduction in mean periodontal parameters at T2 and T3. Overall, the achievement of EoT at T1 was 67.6%, EoT at T2 was 79.2%, while EoT at T3 was 81.3% (Table 3). In the multilevel regression analyses, the factors which negatively influenced EoT at T2 and T3 were multirooted teeth, furcation involvement (degree 2 and 3), initial PPD and interproximal site location (Table 4).

**Table 2:** Effect of step I-II of periodontal therapy and RSI on clinical parameters at different time-points (patient-level)

Variables	T0	T1*	T2*	T3*
FMPS % (mean ± SD)	73.9 ± 15.8	22.3 ± 17.1	16.9 ± 10.0	16.7 ± 10.4
FMBS % (mean ± SD)	63.5 ± 23.0	23.7 ± 16.2	15.1 ± 9.4	14.1 ± 8.2
PPD (mean ± SD)	4.0 ± 1.7	3.1 ± 1.4	3.0 ± 1.3	2.9 ± 1.2
CAL (mean ± SD)	5.2 ± 2.5	4.4 ± 2.3	4.4 ± 2.3	4.3 ± 2.2
REC (mean ± SD)	1.5 ± 1.7	1.7 ± 1.8	1.8 ± 1.8	1.8 ± 1.7
n PPD = 4 mm (mean ± SD)	23.9 ± 14.1	17.8 ± 9.1	15.6 ± 9.2	14.0 ± 9.8
n PPD = 5 mm (mean ± SD)	28.5 ± 15.5	16.6 ± 13.0	13.3 ± 10.8	12.6 ± 11.4
n PPD = 6 mm (mean ± SD)	13.7 ± 10.1	4.4 ± 4.3	2.6 ± 3.2	1.6 ± 2.1
n PPD ≥ 7 mm (mean ± SD)	10.7 ± 7.9	3.4 ± 2.9	2.2 ± 2.7	1.4 ± 1.6

*Abbreviations*: *CAL* clinical attachment level, *FMBS* full-mouth bleeding score, *FMPS* full-mouth plaque score, *PPD* probing pocket depth, *REC* recession, *SD* standard deviation, *T0* baseline, *T1* 4–6 weeks from step II, *T2* 3 months from step II, *T3* 6 months from step II

^*^, all values significantly differed from T0

**Table 3:** Endpoint of therapy (A) and relapse after EoT (B) at different time-points (site-level)

A. Endpoint of Therapy (EoT)	T1	T2	T3
Open pockets at T0 (*n* = 1,450*)	EoT T0-T1	EoT T0-T2	EoT T0-T3
	980 (67.6)	1,148 (79.2)	1,179 (81.3)
	Residual pockets at T1 (*n* = 470)	EoT T1-T2	EoT T1-T3
	PPD ≥ 6 mm (*n* = 172)	57 (33.1)	79 (45.9)
	PPD 4–5 mm BoP + (*n* = 298)	199 (66.8)	215 (72.1)
B. Relapse after EoT		T2	T3
	Closed pockets at T1 (*n* = 980)	Relapse T1-T2	Relapse T1-T3
		88 (9.0)	95 (9.7)

*Abbreviations*: *EoT* endpoints of therapy; open pocket, PPD > 4 mm or PPD = 4–5 mm BoP + , *PPD* probing pocket depth, *NST* non-surgical treatment, *T0* baseline, *T1* 4–6 weeks from step II, *T2* 3 months from step II, *T3* 6 months from step II

^*^73 pockets were on teeth later extracted

**Table 4:** Baseline (T0) factors associated with achievement of the endpoints of therapy in multilevel logistic regression models at T1, T2 and T3

	T1			T2			T3		
Model	Odds ratio	95% CI	*p* value	Odds ratio	95% CI	*p* value	Odds ratio	95% CI	*p* value
Tooth type (multi- vs. single-rooted)	0.63	0.42–0.96	0.030	0.39	0.23–0.65	< 0.001	0.30	0.18–0.49	< 0.001
FI at T0 (no FI as ref.)									
I degree	1.45	0.43–4.84	0.549	3.01	0.47–19.19	0.242	0.76	0.21–2.70	0.669
II degree	0.35	0.15–0.82	0.006	0.36	0.14–0.89	0.028	1.21	0.48–3.04	0.693
III degree	2.10	0.19–23.74	0.623	0.28	0.25–3.07	0.296	0.50	0.04–6.09	0.031
Site location (interproximal vs. buccal/lingual)	0.62	0.39–0.96	0.033	0.44	0.25–0.78	0.005	0.40	0.22–0.72	0.002
PPD at T0 (each mm)	0.49	0.43–0.57	< 0.001	0.49	0.42–0.57	< 0.001	0.49	0.42–0.57	< 0.001
Intercept	306.25	113.19–828.59	< 0.001	1480.45	448.91–4883.39	< 0.001	2016.21	586.21–6934.57	< 0.001

*Abbreviations*: *CI* confidence interval, *FI* furcation involvement, *PPD* probing pocket depth, *T0* baseline, *T1* 4–6 weeks from step II, *T2* 3 months from step II, *T3* 6 months from step I

### RSI in sites PPD ≥ 6 mm at T1

After the first cycle of RSI, 33.1% of sites with residual PPD ≥ 6 mm achieved EoT. At the end of the second cycle, EoT reached up to 45.9% (Table 3). Multilevel analyses indicated that PPD at T1 was the preeminent predictor of EoT at T2 and T3 (Table 5).

**Table 5:** Factors at T1 associated with achievement of the endpoints of therapy in multilevel logistic regression models in sites with PPD ≥ 6 mm and 4–5 mm BoP + after RSI at T2 and T3

	T2			T3		
Model	Odds ratio	95% CI	*p* value	Odds ratio	95% CI	*p* value
Sites 4–5 mm BoP +						
Tooth type (multi- vs. single-rooted)	0.43	0.18–0.99	0.048	0.43	0.20–0.92	0.029
PPD at T1 (5 vs 4 mm)	0.25	0.11–0.57	0.001	0.28	0.13–0.60	0.001
Intercept	11.66	3.93–34.59	< 0.001	11.48	4.55–28.96	0.000
Sites ≥ 6 mm						
Tooth type (multi- vs. single-rooted)	0.69	0.28–1.68	0.410	0.72	0.20–2.59	0.616
PPD at T1 (7 vs 6 mm)	0.14	0.05–0.42	< 0.001	0.55	0.18–1.73	0.308
PPD at T1 (8 vs 6 mm)	0.10	0.02–0.52	0.006	0.12	0.02–0.84	0.033
Intercept	1.29	0.58–2.85	0.531	1.47	0.50–4.35	0.483

*Abbreviations*: *CI* confidence interval, *PPD* probing pocket depth, *T0* baseline, *T1* 4–6 weeks from step II, *T2* 3 months from step II, *T3* 6 months from step I

### RSI in sites PPD 4–5 mm with BoP at T1

After the first cycle of RSI, 66.8% of sites with PPD 4–5 mm with BoP achieved EoT, whereas a second cycle provided slight changes—72.1% (Table 3). Multilevel analyses indicated how multirooted teeth were associated with inferior rates of EoT both at T2 and T3 (OR = 0.43 and OR = 0.43, respectively); as well as sites with PPD = 5 mm (OR = 0.25 and OR = 0.28, respectively) (Table 5).

### Relapse during SPC in sites which achieved EoT at T1

Of the 980 EoT sites at T1, 88 showed relapse at T2 (9.0%), whereas 95 at T3 (9.7%). Multilevel analyses indicated how multirooted teeth (OR = 2.06), PPD at T1 (OR = 0.80), III degree FI (OR = 10.28), and site location (OR = 5.48) were associated with higher rates of relapse at T2; whereas multirooted teeth (OR = 3.08) and site location (OR = 2.23) were associated with higher rates of relapse at T3 (Supplementary Table 1).

### Need for surgeries and cost analysis

After steps I-II, 172 sites had PPD ≥ 6 mm which would have been reflected in 2.5 ± 1.6 surgeries in 24 patients, with a mean associated cost of 1,872.00 ± 1,149.00 €. After the first cycle of RSI, 115 sites had PPD ≥ 6 mm which would have been reflected in 1.7 ± 1.3 surgeries in 23 patients, with a mean associated cost of 1,289.00 € ± 937.00 €. After the second cycle of RSI, 93 sites had PPD ≥ 6 mm which would have been reflected in 1.3 ± 1.3 surgeries in 19 patients, with a mean associated cost of 996.00 € ± 978.00 €. The mean number of surgeries and associated costs significantly decreased at different time-points (Table 6).

**Table 6:** Surgeries planned and analysis of costs after re-evaluation

Variables	T1	T2	T3
N of planned surgeries (mean ± SD)	2.5 ± 1.6	1.7 ± 1.3*	1.3 ± 1.3**
Total planned costs (mean ± SD)	1544 ± 905	1171 ± 721	1024 ± 752
Cost of planned surgeries (mean ± SD)	1488 ± 903	1008 ± 767*	754 ± 731**

*Abbreviations*: *SD* standard deviation, *T0* baseline, *T1* 4–6 weeks from step II, *T2* 3 months from step II, *T3* 6 months from step II

^*^p < 0.05 compared to T1

^**^p < 0.01 compared to T1

## Discussion

In residual PPD ≥ 6 mm, one and two cycles of RSI yielded EoT in one third and 45% of the cases, respectively. When re-instrumenting PPD 4–5 mm BoP + as per EFP guideline, a relevant benefit (two thirds of additional EoT) was found after the first cycle of RSI, while a further cycle only provided minimal additional benefits. Moreover, 10% of the sites which achieved EoT after steps I-II of therapy manifested relapse at T3. PPD at T1, tooth type (multi-rooted) and interproximal site location were significant negative predictors. Finally, number of needed surgeries and associated costs decreased after each cycle of RSI.

The first question of the present study was focused on evaluating the efficacy of RSI in residual PPD ≥ 6 mm, which are recommended for surgery [6]. Our results indicated benefits in performing one or two cycles of RSI in terms of achievement of EoT. This finding partially contrasts with early randomized clinical trials of Badersten et al. [21] and König et al. [22], where the effects of non-surgical therapy were maximized at the first instrumentation (step II), and then they tended to stabilize after the following RSI cycles. Besides some differences in methodology and study population (i.e. including only single-rooted teeth for the former and only aggressive forms of periodontitis in the latter), the aforementioned studies employed mean patient-level differences in periodontal parameters as study outcomes. In the present investigation, EoT thresholds as from EFP guideline were employed, as they are more closely related to the goal of therapy than commonly reported mean differences in fractions of a millimeter [6, 23]. Also, these composite surrogate outcomes are directly transferable to daily practice and easy to interpret for patients and clinicians [24]. Indeed, the present investigation confirmed previous understanding on the effect of RSI on mean differences in clinical parameters (PPD changes, CAL gain, REC increase), while finding significant benefits, albeit partial, on the number of residual pockets after each RSI cycle. According to our findings, clinicians may consider residual PPD at T1 as the most relevant parameter for decision making, since PPD = 6 mm was associated with higher odds of EoT compared to higher PPD values. The rationale for giving ‘more chances’ to RSI stems from classic studies which compared it to surgery, with unclear clinical relevance of the statistical superiority of the latter [8, 25, 26]. Moreover, leaving more time for tissue healing allows the clinician to possess the best semiotic elements for surgical treatment planning and management [27]. Indeed, in our study 81.3% of baseline pockets achieved EoT at 6 months. Therefore, while RSI could not be considered resolutive in all residual PPD ≥ 6 mm, its advantages could be more relevant from the patient’s perspective, in light of the associated reduced biological (less invasiveness) and economic costs related to surgery.

The second question of the present study was focused on testing the effect of RSI in residual PPD 4–5 mm with BoP, as per EFP guideline [6]. The findings from the present study partially confirmed the benefits of RSI in those cases, however the EoT was not achieved in all situations. According to our findings, clinicians may consider residual PPD = 5 mm and multi-rooted teeth as relevant predictors for decision making, since they were associated with lower EoT rates. These predictors are consistent with the available evidence [15, 28]. To this regard, the concept of ‘critical probing depth’ introduced by Lindhe et al. [29] can be advocated, assuming that the surgical approach resulted in more attachment loss than the non-surgical modality when performed in more shallow PPDs and in anterior compared to posterior teeth [30].

The third question was focused on exploring the effect of SPC in clinical sites which achieved EoT after steps I-II, as per the EFP guideline. Our results highlighted a relapse in 10% of those sites, with a higher rate in PPD = 4 mm, interproximal site location and multi-rooted teeth. This observation may be relevant in tailoring SPC recall frequency and protocols [31].

This study presents some limitations, being relatively short-termed, having a sample size which might have reduced the power for patient level predictors (i.e., smoking, diabetes, and lifestyle factors [32–35]) and a monocentric setting which might have affected generalizability. In the present investigation, only light smokers were included (< 10 cigarettes a day), representing a considerable part of the sample (40%). Smoking is known to yield a significant impact on bleeding rates and pocket closure [11], although this variable could not be included in the multilevel analysis. Moreover, it may be also advocated that 4–6 weeks represent a too short healing time before the first re-evaluation. This timing has always represented a matter of debate, the decision being influenced by both biological and clinical observations. Despite the junctional epithelium re-establishes on the tooth surface in 1–2 weeks after scaling and root planing [36], the minimal interval allowed for probing a previously treated site is considered 4 to 6 weeks, being the connective tissue slower to heal [37]. However, since clinical guidelines do not make any specific recommendation about this matter, we have based our choice according to the aforementioned biological rationale and to previously published articles [38]. Finally, the absence of a control group could not allow speculation on whether some sites would have closed anyway even without re-instrumenting, or whether surgery would have been more effective in closing pockets than RSI at both short- and long-term. Future randomized clinical trials may clarify all these clinically relevant aspects, providing practice-based evidence feedback for the most effective real-life application of the available EFP guideline.

## Conclusions

The current study suggests that RSI may achieve EoT in both residual PPD 4–5 mm BoP + and PPD ≥ 6 mm in a considerable number of cases. PPD at T1, tooth type (multi-rooted) and interproximal site location were significant negative predictors of EoT. Moreover, the number of needed surgeries and associated costs significantly decreased after each RSI cycle. Despite study limitations, the present findings support the decision to perform one or two cycles of RSI before evaluating the surgical approach, for both biological and economical cost justification.

### Supplementary Information

Below is the link to the electronic supplementary material.

Supplementary file1 (DOCX 18 KB)

## Data Availability

The data that support the findings of this study are available from the corresponding author upon reasonable request.
