# Supplementary material for: Pocket closure after repeated subgingival instrumentation: a stress test to the EFP guideline for stage III-IV periodontitis
Source: Clin Oral Investig. 2023 Sep 29;27(11):6701–8. doi: 10.1007/s00784-023-05279-6 (PMC10630226; doi:10.1007/s00784-023-05279-6)
Supplement: Supplementary file 1 — Supplementary file1 (DOCX 18 KB) [file 784_2023_5279_MOESM1_ESM.docx]

**Supplementary Table 1.** Factors at T1 associated with relapse after EoT in multilevel logistic regression models at T2 and T3

|  | **T2** | | | **T3** | | |
| --- | --- | --- | --- | --- | --- | --- |
| **Model** | **Odds ratio** | **95% CI** | **p value** | **Odds ratio** | **95% CI** | **p value** |
| Tooth type (multi- vs. single-rooted) | 2.06 | 1.21-3.51 | 0.008 | 3.08 | 1.86-5.10 | <0.001 |
| PPD at T1 (each mm) | 0.80 | 0.65-0.99 | 0.039 | 0.83 | 0.68-1.00 | 0.053 |
| FI at T0 (no FI as ref.) |  |  |  |  |  |  |
| I degree | 2.98 | 0.83-10.66 | 0.093 | 0.63 | 0.13-3.04 | 0.563 |
| II degree | NE | NE | NE | 0.49 | 0.06-4.33 | 0.524 |
| III degree | 10.28 | 1.23-86.22 | 0.032 | 3.85 | 0.34-43.85 | 0.277 |
| Site location (interproximal vs. buccal/lingual) | 5.48 | 1.87-16.05 | 0.002 | 2.23 | 1.03-4.87 | 0.043 |
| Intercept | 0.01 | 0.00-0.05 | <0.001 | 0.03 | 0.01-0.08 | <0.001 |

**Abbreviations:** CI, confidence interval; FI, furcation involvement; NE, not estimable; PPD, probing pocket depth; T1, 1 month from step II; T2, 3 months from step II; T3, 6 months from step II.
